# Role of Muscle Mass and Nutritional Assessment Tools in Evaluating the Nutritional Status of Patients With Locally Advanced Nasopharyngeal Carcinoma

**DOI:** 10.3389/fnut.2021.567085

**Published:** 2021-03-08

**Authors:** Xi Pan, Hong Liu, Guo Feng, Jie Xiao, Meng Wang, Hua Liu, Xueyi Xie, Zhipeng Rong, Jinru Wu, Min Liu

**Affiliations:** ^1^Department of Oncology, The Third Xiangya Hospital, Central South University, Changsha, China; ^2^Department of Nutrition, The Third Xiangya Hospital, Central South University, Changsha, China; ^3^Department of Central Sterile Supply, The Third Xiangya Hospital, Central South University, Changsha, China

**Keywords:** body mass index, fat-free mass index, nasopharyngeal carcinoma, body composition analysis, nutritional status assessment

## Abstract

**Objective:** This study was to explore the role and necessity of muscle mass [fat-free mass index (FFMI) and appendicular skeletal muscle index (ASMI) measured by bioelectrical impedance analysis (BIA)] in nutritional status evaluation of patients with locally advanced (III, IVa) nasopharyngeal carcinoma (NPC).

**Methods:** One hundred and thirty locally advanced NPC patients were recruited. Their nutritional status was assessed by albumin (ALB), body mass index (BMI), Nutritional Risk Screening 2002 (NRS 2002), Patient generated-Subjective Global Assessment (PG-SGA), and muscle mass. Consistency test and McNemar test were used to evaluate the consistency of muscle mass with ALB, BMI, NRS 2002, and PG-SGA, and correlation analysis was performed on muscle mass and PG-SGA or BMI.

**Results:** 61/130 (46.9%) of the patients had nutritional risks according to NRS 2002, 68/130 (53.1%) of the patients had malnutrition according to PG-SGA assessment. FFMI and ASMI could determine the loss of muscle mass that cannot be detected by albumin (30.2 and 65.6%), BMI (28.0 and 35.3%), NRS 2002 (26.1 and 25.0%), and PG-SGA (18.6 and 55.6%). McNemar test showed that the malnutrition results assessed by FFMI and BMI were inconsistent (*P* <0.001), but further Pearson correlation analysis showed that BMI was positively correlated with FFMI (*rs* = 0.300, *P* = 0.001).

**Conclusion:** The commonly used nutritional assessment scale/parameters cannot identify the muscle mass loss in patients with locally advanced NPC. Analysis of human body composition is important for nutritional assessment in patients with locally advanced NPC.

## Introduction

Nasopharyngeal carcinoma (NPC) is a type of malignant tumor originated from nasopharynx and is one of the most common head and neck malignant tumors in China (1). Due to the concealed anatomy of the nasopharynx, the early symptoms of the patients are not obvious, and our hospital found that 70% of the 1,200 patients present with locally advanced stages (stage III and IVa) at the time of diagnosis. Besides the tumor burden, NPC patients would be affected simultaneously by side effects of chemoradiotherapy and sequence radiotherapy, and would often be accompanied by varying degrees of nutritional risk or malnutrition, such as wasting, anemia, and hypoproteinemia, which may directly affect the control and prognosis of the tumor (2–4). Therefore, early detection of the NPC patients at nutritional risk, timely nutritional treatment may reduce the incidence of malnutrition and may improve the effectiveness of cancer treatment.

Expert Consensus on nutrition therapy for head and neck cancer in Mainland and Taiwan China, and the United Kingdom recommended either Nutritional Risk Screening 2002 (NRS 2002) as a nutritional risk screening tool or Patients Generated-Subjective Global Assessment (PG-SGA) as a nutritional assessment tool (5, 6). Body mass index (BMI) and albumin (ALB) are also nutritional assessment scales or parameters currently used in patients with NPC.

But the commonly used nutritional assessment scale/parameters cannot recognize the muscle loss, and the harm of muscle mass attenuation in patients with locally advanced NPC has not been valued in China. The European Society of Clinical Nutrition and Metabolism (ESPEN) suggested reduced fat-free mass index (FFMI) combined with %weight loss [alternatively, BMI < 20 kg/m^2^ (<70 years)/<22 kg/m^2^ (70 or older)] as one of the two alternative criteria to diagnose malnutrition (7). Asian working groups for sarcopenia 2019 recommended appendicular skeletal muscle mass index (ASMI) as an indicator for muscle mass (8). Patients with malignant tumors, such as liver cancer, gastric cancer, colorectal cancer, and head and neck cancer, usually have muscle mass decreases, which will affect the outcome of the diseases (8–11). Presence of low ASMI + low muscle strength or low physical performance was identified as independent predictor of reduced overall survival among cirrhotic patients with hepatocellular carcinoma (12).

This study was to investigate the consistency of FFMI and ASMI (measured by Bioelectrical impedance Analysis, BIA) with the commonly used nutritional assessment scale/parameters (NRS 2002, PG-SGA, BMI, and ALB), and to explore the effect of body composition analysis on nutritional assessment in patients with locally advanced NPC.

## Materials and Methods

### Subjects

This study recruited 130 patients with locally advanced NPC diagnosed from June 2018 to October 2019. Informed consent was signed by all of the recruited patients. The inclusion criteria were: (1) age 18–90 years old, (2) with newly diagnosed NPCs according to the diagnostic criteria, (3) the patients had no more than 10 times of radiotherapy and no more than three times of chemotherapy, (4) no serious ascites and edema, and (5) no surgery before 8 a.m. the next day and the hospital stay was more than 24 h.

### Methods

The InBodyS10 analyzer, a multifrequency BIA (Biospace Co., Ltd., Seoul, Korea) was used to estimate the human body composition. InBody uses an eight-point quadrupole electrode system method to evaluate the impedance of small alternating current applied to the body at three specific frequencies (5, 50, and 250 kHz) and six specific frequencies (1, 5, 50, 250, 500, and 1,000 kHz). The BIA measurements were conducted by trained personnel in accordance with the standardized procedures.

The enrolled patients were firstly diagnosed with nasopharyngeal carcinoma and had no more than 10 times of radiotherapy and no more than three times of chemotherapy. BIA was measured when patients were firstly enrolled. Patients had an overnight fast, emptied the bladder by urinating, took off the clothes, and kept a standing posture during the measurement, during which the ambient temperature remained at 25°C.

FFMI = fat free mass/(height × height). FFMI of <17 kg/m^2^ for men or <15 kg/m^2^ for women was defined as low FFMI (+) according to the cut-off values for FFMI by ESPEN (7). ASMI = appendicular skeletal muscle mass/(height × height). ASMI of <7 kg/m^2^ for men or <5.7 kg/m^2^ for women was defined as low FFMI (+) according to the cut-off values for ASMI by Asian-based reference (8).

Height and weight were measured at fasting status and shoes-free, respectively, and the BMI was calculated. BMI < 18.5 kg/m^2^ indicates malnutrition (BMI +). Albumin was measured by fasting blood, Albumin <30 g/L was considered low Albumin (Albumin +). Nutritional risk was screened by NRS 2002, and a NRS 2002 score ≥3 was a suggestive of nutritional risk (NRS 2002 +) (13). PG-SGA was used for nutritional assessment, and PG-SGA score ≥4 indicated malnutrition (PG-SGA +) (14).

### Statistical Analysis

SPSS version 17.0 (Statistical Product and Service Solutions, CA, USA) was used for statistical analysis. NRS 2002, PG-SGA, BMI, albumin, ASMI and FFMI were subjected to normal distribution test. Normally distributed variables (BMI, albumin, ASMI and FFMI) were expressed as mean ± standard deviation (SD); NRS 2002 was classified as 3, 4, and 5 scores. PG-SGA was classified as 0–3 scores, 4–8 scores, and ≥9 scores. Use McNemar test and consistency test analysis to perform consistency on NRS 2002, PG-SGA, BMI, or albumin with FFMI or ASMI. Pearson correlation analysis was used between FFMI or ASMI and BMI. A value of *P* < 0.05 was considered statistically significant.

## Results

### General Characteristics

One hundred and thirty locally advanced NPC patients were recruited into the study, 67.7% of whom were men, the mean age is 49 ± 11.3 years old. The tumor, node and metastasis (TNM) stages were ranging from III to IVa periods. The characteristics of patients and tumor are shown in Table 1.

**Table 1:** Baseline characteristics of study sample (*n* = 130).

**Characteristics**	**Number of patients (%)**	**Characteristics**	**Number of patients (%)**
Sex		% of non-volitional weight loss during 6 months	
Male	88 (67.7%)	<5%	94 (72.3%)
Female	42 (33.3%)	Between 5 and 10%	26 (20.0%)
Age (years) mean ± SD	49 ± 11.3	More than 10%	10 (7.7%)
TNM stage (AJCC 2007)		Stage of treatment (Radiotherapy)	
I	(0.0%)	0–10 times	130 (100.0%)
II	(0.0%)	0 times	34 (26.2%)
III	80 (61.5%)	1–3 times	37 (28.5%)
IVA	50 (38.5%)	4–7 times	38 (29.2%)
		8–10 times	21 (16.1%)
Anorexia	0 (0.0%)	Stage of treatment (chemotherapy)	
Food intake		0–3 times	130 (100.0%)
Sufficient	21 (16.2%)	0 times	40 (30.8%)
Insufficient	109 (83.8%)	1 time	28 (21.5%)
		2 time	36 (27.7%)
		3 time	26 (20.0%)
Seriously inadequate	21 (19.3%)	Nutrition therapy at the time of enrollment	
Moderately inadequate	39 (35.8%)	Yes	0 (0.0%)
Slightly inadequate	49 (44.9%)	No	130 (100.0%)

*TNM, tumor, node, and metastasis; AJCC, American Joint Committee on Cancer; Seriously inadequate: Intake less than half of targets; Moderately inadequate: Intake was between half and three quarters of targets; Slightly inadequate: Intake more than three quarters of targets*.

The mean FFMI was 17.2 ± 1.7 kg/m^2^. Of them, 30.8% had low FFMI, including 27 males and 13 females. The mean ASMI was 7.24 ± 0.54 kg/m^2^. Of them, 35.4% had low ASMI, including 39 males and 7 females. Only 3.8% of the patients had a BMI lower than 18.5 kg/m^2^ (including three males and two females), and the mean BMI was 22.5 ± 2.3 kg/m^2^. Only 0.8% of the patients had an albumin lower than 30 g/L, and the mean albumin was 40.0 ± 3.3 g/L (Table 2).

**Table 2:** Nutritional status and human body composition of locally advanced NPC patients (*n* = 130).

**Parameters**	**± s or M ± QR**
Height (cm), mean ± SD	163.5 ± 9.00
Weight (kg), mean ± SD	60.00 ± 9.47
FFMI (kg/m^2^)	17.21 ± 1.65
ASMI (kg/m^2^)	7.24 ± 0.54
BMI (kg/m^2^)	22.54 ± 2.28
Albumin	40.05 ± 3.28
**NRS 2002 score**	
3	43 (33.1%)
4	15 (11.5%)
5	3 (2.3%)
**PG-SGA score**	
0–3	61 (46.9%)
4–8	37 (28.5%)
≥9	32 (24.6%)

*± s refers to mean ± standard deviation (SD), or M ± QR refers to median ± quartile range*.

*FFMI, fat-free mass index; ASMI, appendicular skeletal muscle mass; BMI, body mass index; NRS2002, nutrition risk screening 2002; PG-SGA, Patient Generated Subjective Global Assessment*.

Almost half (46.9%) of the patients had nutritional risk when assessed with NRS 2002 (NRS 2002 ≥3 scores). The patients with a score of 3 were 43 cases (33.1%), a score of 4 were 15 cases (11.5%), and a score of equal or more than 5 were three cases (2.3%). An assessment with PG-SGA showed 53.1% of the patients had suspected malnutrition (PG-SGA ≥4 scores). The patients with a score of 0–3 were 61 cases (46.9%), a score of 4–8 were 37 cases (28.5%), and a score of equal or more than 9 were 32 cases (24.6%) (Table 2).

### Baseline Characteristics by Age Stratification

The mean age of our patients was 49 ± 11.3 years old. We analyzed the FFMI, BMI and albumin of the patients by age stratification and found that FFMI was lower in patients with lower BMI. We found FFMI a growing polarization of trends with age. FFMI is lower in patients with age <40 years or older than 60 years (Table 3). We also analyzed the ASMI, BMI and albumin of the patients by age stratification and found that ASMI was lower in older patients. We found ASMI has no correlation with BMI (Table 3).

**Table 3:** Baseline characteristics of study sample stratified by age and % weight loss (*n* = 130).

**Age stratification (years old)**	**Number (%)**	**FFMI (kg/m^**2**^)**	**ASMI (kg/m^**2**^)**	**BMI (kg/m^**2**^)**	**Albumin (g/L)**
≤30	6 (4.6%)	14.6 ± 0.55	7.69 ± 0.34	18.9 ± 2.55	41.1 ± 3.32
31–40	11 (8.5%)	15.9 ± 1.72	7.35 ± 0.25	20.4 ± 1.49	41.4 ± 3.76
41–50	55 (42.3%)	17.8 ± 0.98	7.31 ± 0.86	23.3 ± 1.64	40.6 ± 2.55
51–60	42 (32.3%)	17.6 ± 1.21	7.32 ± 0.44	23.2 ± 2.12	40.1 ± 3.10
61–70	16 (12.3%)	15.8 ± 1.55	6.57 ± 0.62	20.7 ± 0.67	37.8 ± 1.74
**% of weight loss**					
<5%	94 (72.3%)	16.1 ± 0.84	7.20 ± 1.59	22.7 ± 2.56	40.4 ± 2.75
Between 5 and 10%	26 (20.0%)	17.6 ± 1.98	7.17 ± 1.24	21.7 ± 1.45	40.3 ± 3.67
More than 10%	10 (7.7%)	14.8 ± 2.21	7.83 ± 0.85	23.1 ± 2.04	37.9 ± 0.96

*FFMI, fat-free mass index; ASMI, appendicular skeletal muscle mass; BMI, body mass index*.

In addition, we added % of weight loss in Table 3 and found that FFMI was lowest on average among those who had lost more than 10% of their body weight in the last 6 months (FFMI 14.8 ± 2.21 kg/m^2^) (Table 3).

### Consistency Between the Results of Human Body Composition and the Results of Nutritional Assessment Scales/Parameters

There was consistency between FFMI and BMI in the assessment of malnutriton (*P* = 0.001) but the consistency was poor (Kappa = 0.165). The rate of low FFMI was 30.8%, significantly higher than that of low BMI (3.8%), and the difference was statistically significant (*P* < 0.001). Inconsistency was noted between FFMI and albumin, FFMI and NRS 2002, FFMI and PG-SGA in the assessment of malnutrition (*P* = 0.132, 0.219, and 0.501, separately). The rate of low FFMI was 30.8%, significantly higher than that of low albumin (0.8%), and the difference was statistically significant (*P* < 0.001) (Table 4).

**Table 4:** Consistency of the fat-free mass index (FFMI) and appendicular skeletal muscle mass (ASMI) with the nutritional scales/parameters in the nutritional assessment (*n* = 130).

**BIA**		**BMI**		**Albumin**		**NRS2002**		**PG-SGA**		**Total**
		**+**	**–**	**+**	**–**	**+**	**–**	**+**	**–**	
FFMI	+	5 (100.0)	35 (28.0)	1 (100.0)	39 (30.2)	22 (36.1)	18 (26.1)	23 (32.4)	17 (18.6)	40 (30.8)
	–	0 (0.0)	90 (72.9)	0 (0.0)	90 (69.8)	39 (63.9)	51 (73.9)	46 (67.6)	44 (81.4)	90 (69.2)
ASMI	+	0 (0.0)	46 (36.8)	0 (0.0)	46 (35.7)	28 (45.9)	18 (26.1)	23 (32.4)	23 (37.7)	46 (35.4)
	–	5 (100.0)	79 (63.2)	1 (100.0)	83 (64.3)	33 (54.1)	51 (73.9)	46 (67.6)	38 (62.3)	84 (64.6)
Total		5 (3.8)	125 (96.2)	1 (0.8)	129 (99.2)	61 (46.9)	69 (53.1)	69 (53.1)	61 (46.9)	130 (100.0)

*FFMI, fat-free mass index; ASMI, appendicular skeletal muscle mass; BMI, body mass index; NRS2002, nutrition risk screening 2002; PG-SGA, Patient Generated Subjective Global Assessment*.

**Figure 1:**
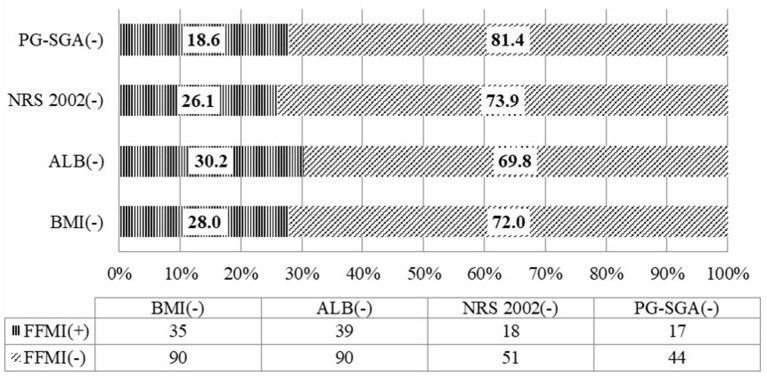
Consistency of the fat-free mass index with the nutritional scales/parameters in the nutritional assessment of locally advanced NPC patients (*n* = 130).

Among the patients whose nutritional risk screening by NRS 2002 was lower than the score of 3, the rate of low FFMI was 26.1%. Among the patients without malnutrition assessed by PG-SGA, the rate of low FFMI was 18.6%. And 28% of patients whose BMI was equal or more than 18.5 kg/m^2^ have low FFMI. Among patients with albumin equal or more than 30 g/L, the rate of low FFMI was as high as 30.2% (Figure 1).

**Figure 2:**
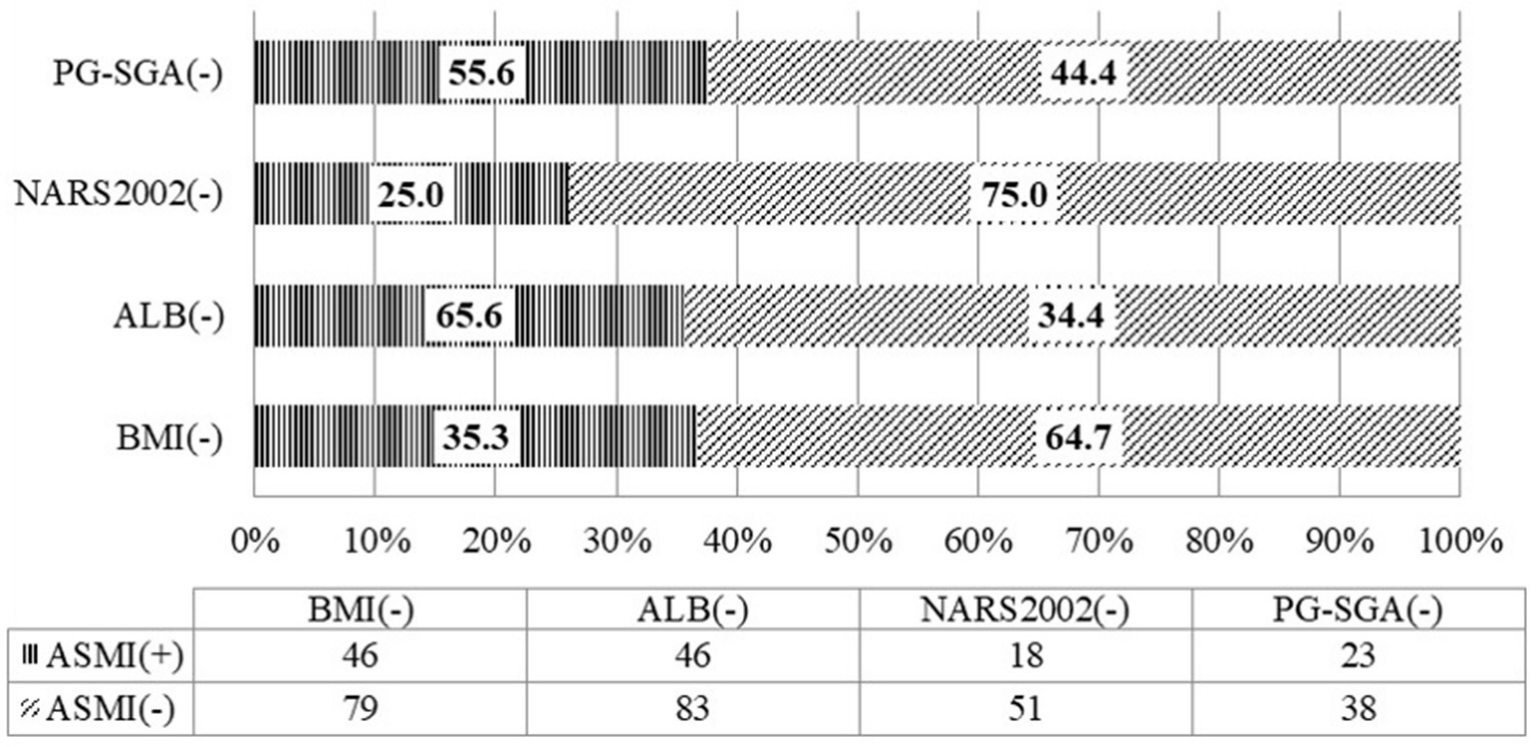
Consistency of the appendicular skeletal muscle mass with the nutritional scales/parameters in the nutritional assessment of locally advanced NPC patients (*n* = 130).

There was consistency between ASMI and BMI in the assessment of malnutriton (*P* = 0.012) but the consistency was poor (Kappa = 0.092). The rate of low FFMI was 35.4%, significantly higher than that of low BMI (3.8%), and the difference was statistically significant (*P* < 0.001). Inconsistency was noted between ASMI and PG-SGA in the assessment of malnutrition (*P* = 0.961) (Table 4). Among the patients whose nutritional risk screening by NRS 2002 was lower than the score of 3, the rate of low ASMI was 25.0%. Among the patients without malnutrition assessed by PG-SGA, the rate of low ASMI was 55.6%. And 35.3% of patients whose BMI was equal or more than 18.5 kg/m^2^ have low ASMI. Among patients with albumin equal or more than 30 g/L, the rate of low ASMI was as high as 65.6% (Figure 2).

### Correlation of the Results of FFMI and ASMI With BMI

**Figure 3:**
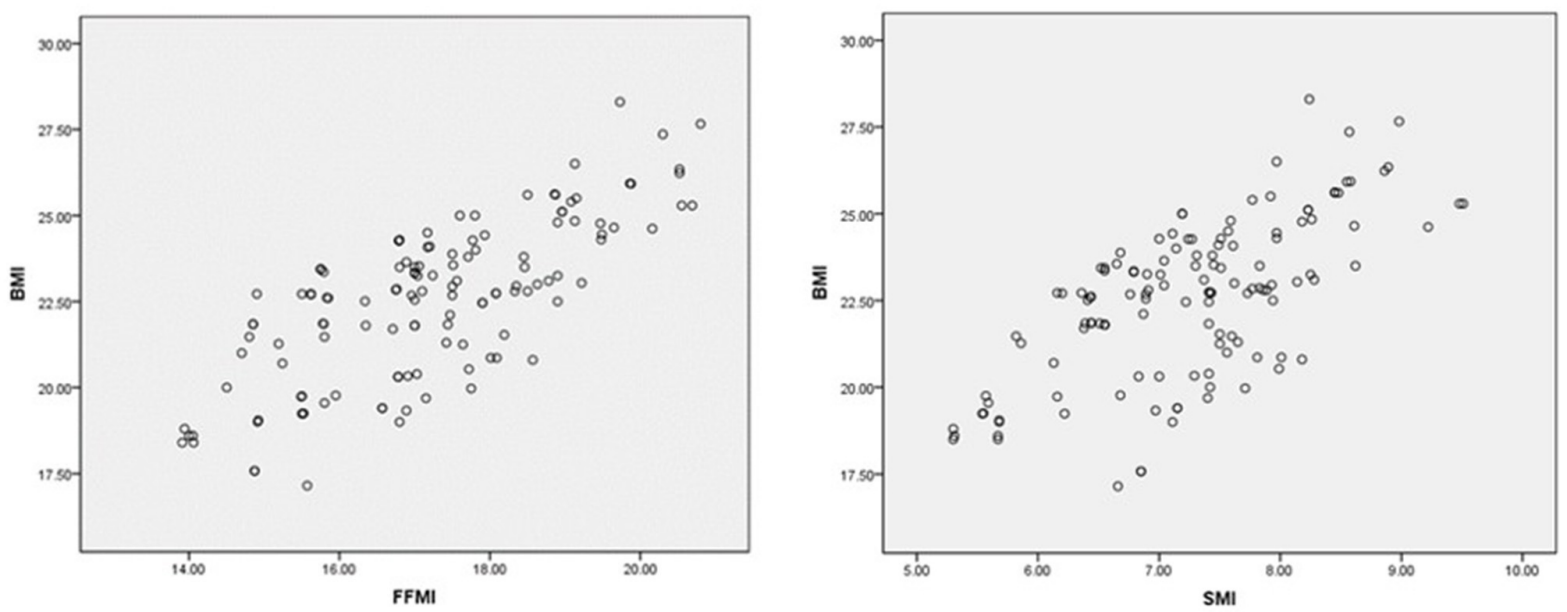
Correlation analysis of FFMI, ASMI, and BMI. The X-axis and Y-axis cut-point was chosen based on the risk level of BMI and BIA parameters.

Consistency was noted among FFMI, ASMI, and BMI in the assessment of nutritional status. A further correlation analysis by Pearson showed that there was a positive correlation between FFMI, ASMI, and BMI (rs = 0.30, *P* = 0.001; and rs = 0.67, *P* < 0.001, separately (Figure 3).

## Discussion

Patients with NPC often face multiple nutritional problems before, during, and after treatment because of the closeness of the cancer to organs that are critical for normal eating function. Dysgeusia, nausea, dysphagia, mucositis, xerostomia, and vomiting are the common treatment-related side effects that further impair the patient's ability to maintain adequate oral intake. Regular assessment of the nutritional status of patients with nutritional risk and early initiation of nutritional therapy can improve the outcomes (15, 16).

ESPEN suggested reduced muscle mass as a selective criterion to diagnose malnutrition (7). The content of muscle mass reduction appeared in Global Leadership Initiative on Malnutrition (GLIM) assessment standard consensus published in 2019 (17, 18). According to the GLIM assessment, malnutrition can be diagnosed when one phenotypic criterion and one etiological criterion are present. The phenotypic criteria include weight loss, low BMI, or reduced muscle mass, and the etiological criteria include assimilation or reduced food intake, disease burden and inflammation (18, 19). More and more studies showed that the reduction of muscle mass and function was associated with a poor prognosis in patients with NPC (20). At present, the commonly used method nutrition screening scales/parameters in China, such as BMI, albumin, NRS 2002, and PG-SGA do not include muscle mass.

FFMI is one of the indexes of muscle mass commonly used in recent years. It has been recognized that the reduction in fat-free mass (FFM) is linked to the poor prognosis of cancer patients (21–25). Reduced FFMI is an independent predictor of post-operative complications, chemotherapy toxicity, and mortality in cancer patients (21–25). ESPEN defined low FFMI as lower than 15 or 17 kg/m^2^ in females or males, respectively (7). Although magnetic resonance imaging (MRI), computed tomography (CT), and dual-energy X-ray absorptiometry (DEXA) are gold standards for evaluating muscle mass, they are expensive or radioactive. Fürstenberg et al. found that bioelectrical impedance analysis (BIA) was highly consistent with DEXA in evaluating muscle mass (26). Moreover, BIA has the advantages of low cost, non-invasive, safe, simple, and fast. Asian working group for sarcopenia supported ASMI measurement using BIA (8). ESPEN also suggested BIA as an objective measurement for fat free mass (FFM) and skeletal muscle mass (ASM) (27). In this study, BIA was used to measure the FFM and ASM in patients with locally advanced NPC. Our study showed that locally advanced NPC patients had poor nutritional status. From these patients, almost half (46.9%) had nutritional risk when screening with NRS 2002, and 53.1% of the patients had malnutrition when assessment with PG-SGA. Only 3.8% of the patients had a BMI lower than 18.5 kg/m^2^, and the average BMI was 22.54 ± 2.28 kg/m^2^. Low FFMI can be detected in 30.8% of the patients. Both assessments with nutritional scales/parameters and human body composition analysis suggest that more than 30% of the patients have nutritional risk or malnutrition. Only 1 patient (0.8%) had hypoproteinemia, showed the importance of albumin is questioned in malnutrition evaluation among locally advanced NPC patients.

Our study also showed that 26.1% of the patients screened as having no nutritional risk by NRS 2002 had low FFMI. This means that if we only use NRS2002, we may neglect a number of patients who had low muscle mass. ESPEN guidelines in 2008 recommend that BMI lower than 18.5 kg/m^2^ combined with poor general status could be assessed as malnutrition. This standard will still be used by many people in China to assess malnutrition. However, we found 28.0% of the patients with normal BMI had low FFMI in our study. We analyzed the FFMI and BMI of the patients by age stratification and found that FFMI was lower in patients with lower BMI. Surprisingly, we found FFMI a growing polarization of trends with age. FFMI is lower in patients with age <40 or older than 60.

Meanwhile, we analyzed the ASMI of the patients by age stratification and found that ASMI was lower in older patients. In addition, every time there is weight loss there is an added increase in the risk of further loss of muscle mass. Further studies showed that FFMI was lowest on average among those who had lost more than 10% of their body weight in the last 6 months (FFMI 14.8 ± 2.21 kg/m^2^).

We also found that among patients with normal nutritional status assessed by PG-SGA, 18.6% had reduced FFMI. Further consistency analysis found that FFMI and NRS 2002, or PG-SGA had a poor consistency. According to the GLIM assessment, reduced muscle mass is an important phenotype criterion for diagnosis of malnutrition. Therefore, it is possible to diagnose malnutrition through body composition analysis in patients with no nutritional risk or normal nutritional status screened by traditional nutrition scale/parameters. Malignant disease patients with normal weight or even obesity accompany depleted muscle mass, might predict higher morbidity, mortality and also higher toxicity to chemotherapy (25, 26). The results suggested that the BMI, nutritional risk screening and nutritional status assessment should be treated flexibly in clinical practice (28). Neither NRS 2002 nor PG-SGA can accurately reflect the muscle mass of patients. Both PG-SGA and NRS 2002 may miss the identification of malnutrition in some patients. Once low muscle mass is present in locally advanced NPC patients, they should receive nutritional support.

## Limitations of the Study

The limitations of our study are the small sample size and the relatively heterogeneous population. Although our study was conducted in the oncology department of our hospital, our patients were from different regions of Hunan province. In the future, more prospective and large sample studies are needed to verify our findings.

## Conclusions

In summary, poor nutritional status and low muscle mass was common in patients with locally advanced NPC. The assessment of malnutrition in locally advanced NPC should be individualized, based on multiple factors, such as nutritional risk screening, nutritional status assessment scales/parameters and body composition analysis.

## Data Availability Statement

The original contributions presented in the study are included in the article/supplementary material, further inquiries can be directed to the corresponding author/s.

## Ethics Statement

The studies involving human participants were reviewed and approved by the Institutional Review Boards of the Third Xiangya Hospital, Central South University (No: 2019-S542), China. The patients/participants provided their written informed consent to participate in this study.

## Author Contributions

ML led the study design and approved the final version of the manuscript. XP, HL, GF, MW, JX, XX, ZR, JW, and ML collected and analyzed the data. XP, HL, and ML drafted the manuscript. All authors have read and approved the manuscript.

## Conflict of Interest

The authors declare that the research was conducted in the absence of any commercial or financial relationships that could be construed as a potential conflict of interest.
